# Triple-phase boundary and power density enhancement in thin solid oxide fuel cells by controlled etching of the nickel anode

**DOI:** 10.1186/1556-276X-9-286

**Published:** 2014-06-09

**Authors:** Rabi Ebrahim, Mukhtar Yeleuov, Ainur Issova, Serekbol Tokmoldin, Alex Ignatiev

**Affiliations:** 1 Center for Advanced Materials, University of Houston, Houston, TX 77204-5004, USA; 2 Department of Physics, University of Houston, Houston, TX 77204-5005, USA; 3 Department of Electrical and Computer Engineering, University of Houston, Houston, TX 77204-4005, USA; 4 Institute of Physics and Technology, Almaty 050032, Kazakhstan

**Keywords:** Thin film, Solid oxide, Fuel cell, Triple-phase boundaries, YSZ, LSCO

## Abstract

Fabrication of microporous structures for the anode of a thin film solid oxide fuel cell (SOFC(s)) using controlled etching process has led us to increased power density and increased cell robustness. Micropores were etched in the nickel anode by both wet and electrochemical etching processes. The samples etched electrochemically showed incomplete etching of the nickel leaving linked nickel islands inside the pores. Samples which were wet- etched showed clean pores with no nickel island residues. Moreover, the sample with linked nickel islands in the anode pores showed higher output power density as compared to the sample with clean pores. This enhancement is related to the enlargement of the surface of contact between the fuel-anode-electrolyte (the triple-phase boundary).

## Background

The world's extensive use of petroleum increased drastically in the last decades causing not only a sharp drop in the world reserves but also resultant environmental concerns. Natural gas and other high hydrogen content fuels are better replacement candidates because of their lower environmental effects [1-3]. The major shortcomings of these types of fuels are their lower combustion efficiency and the larger volumes needed for machines that convert the fuel to electrical energy. This opens the field for more research on the development of low-volume and high-efficiency generators in order to use these fuels in a wide range. Extensive research has been held on fuel cells, which are one of the promising candidates. A number of hydrogen-oxygen-operated fuel cell designs already exist; solid oxide fuel cells (SOFCs) are one of the most attractive fuel cell types due to their high energy efficiency and environmental friendliness [4]. Thick solid oxide fuel cells exhibited 0.2 to 1 W/cm^2^ with 60% to 70% reported efficiency but at undesired high operating temperatures >800°C [5,6]. To avoid the high operating temperature of the SOFCs, it has been proposed to reduce electrolyte thickness and/or use a higher ion conducting electrolyte material. The fabrication of ultra-thin film SOFCs (10- to 15-μm cell thickness) built on microporous thin metallic foil substrates has already shown considerable reduction of the operating temperatures to 450°C to 550°C and also a reduction of cell volume. However, the cell was somewhat structurally weak, and cell output power density was low as compared to known SOFCs [7]. In the present work, the enhancement of both cell physical structure and the output power density have been achieved by controlling the chemical etching process of the thin metal anode micropores which in turn enhanced the surface of contact between fuel-anode-electrolyte (the triple-phase boundary) [5,8].

## Methods

**Figure 1:**
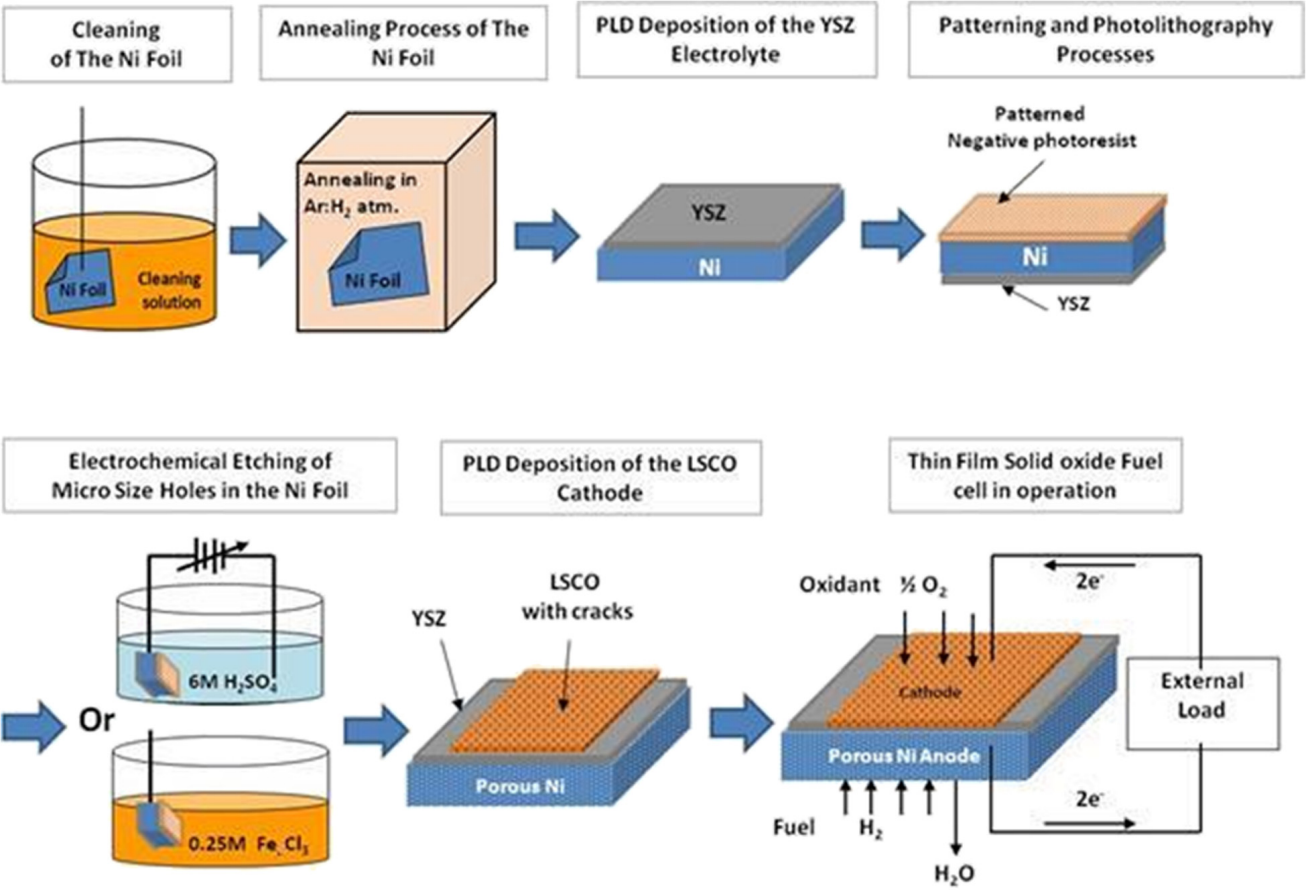
Schematic diagram for LSCO/YSZ/Ni thin SOFC(s) fabrication process flow.

The electrolyte and cathode layers of the thin film SOFCs were fabricated on 10-μm-thick nickel foil (to act as an anode). The thin film solid oxide fuel cell fabrication process flow is illustrated in Figure 1, wherein the nickel foils were treated for a short time in a mixture of acetic, nitric, sulphuric, and phosphoric acids to remove any rolling marks left on the foil surface followed by a degreasing process (acetone, methanol, and DI water). The clean nickel foils were annealed at 650°C for 2 h in an argon atmosphere in order to generate atomic ordering with the lattice (100) direction normal to the foil surface. Layers of yttria-stabilized zirconia (YSZ) electrolyte (approximately 1.5 μm thick) and La_0.5_Sr_0.5_CoO_3 - *δ*_ (LSCO) cathode (approximately 2 μm thick) were deposited on the nickel foils using pulsed laser deposition (PLD; 248-nm KrF laser) in an initially 96% argon/4% hydrogen atmosphere (to avoid nickel oxidization) and then in an oxygen atmosphere (to yield good oxide stoichiometry) at substrate temperatures of 25°C to 650°C. Hexagonal pores (about 50-μm diameter with 50-μm spacing) were etched in the nickel anode by photolithographic patterning followed by either wet etching (using 0.25 M FeCl_3_) or electrochemical etching (using 6 M H_2_SO_4_) at room temperature (see Figure 1).

The crystalline structures of the successive layers of the fabricated fuel cells were characterized by X-ray diffraction (XRD) measurements which were carried out using a Siemens D-5000 spectrometer (Erlangen, Germany). The XRD scans were done in the standard *θ*-2*θ* configuration, using the Cu Kα radiation of wavelength 1.54 Å at scan steps of 0.05°. SEM analysis was carried out using a JEOL (JSM 5410, Akishima, Tokyo, Japan) scanning electron microscope. A computerized testing setup was used to test the fuel cells fuel-air performance (*I*-*V* and power output characteristics) as a function of operating temperature.

## Results and discussion

**Figure 2:**
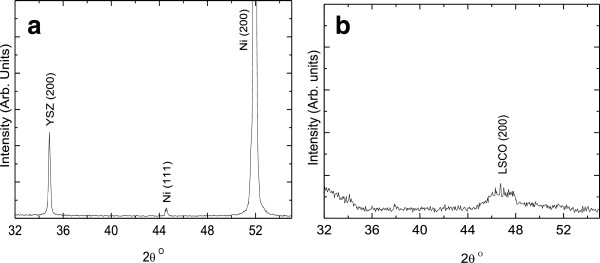
XRD scans for (a) YSZ/Ni and (b) LSCO/YSZ/Ni films deposited by PLD.

**Figure 3:**
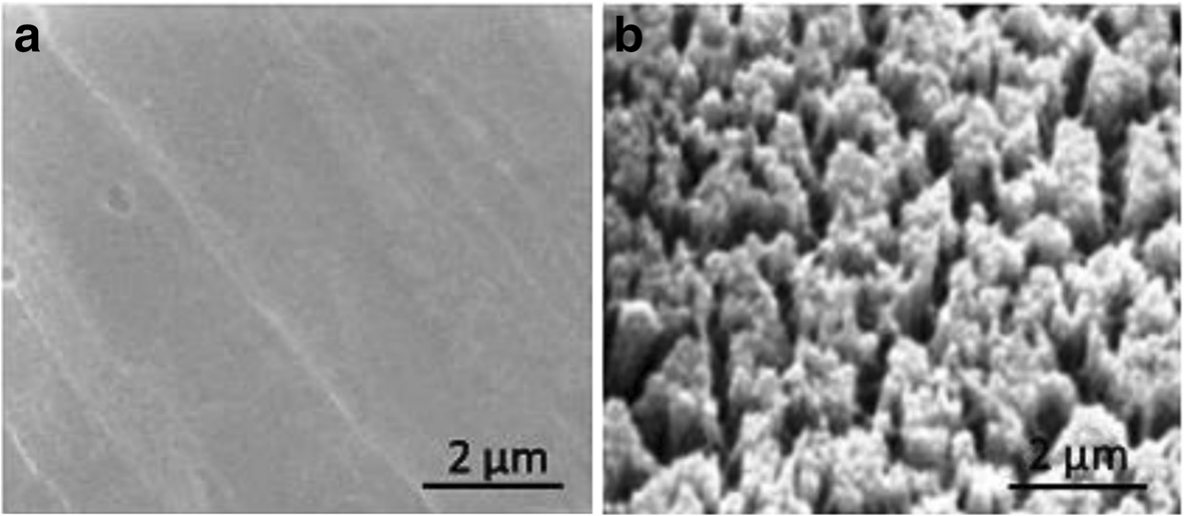
Surface SEM micrographs of thin SOFC layers: (a) YSZ/Ni (uniform electrolyte) and (b) LSCO/YSZ/Ni (cracked cathode).

The XRD scans of the different layers of the fabricated samples are shown in Figure 2. The XRD scan of the approximately 1.5-μm-thick YSZ electrolyte film deposited on treated nickel foil by PLD at 650°C (Figure 2a) shows two major peaks: Ni (200) at *θ* = 51.85° and YSZ (200) at *θ* = 34.8°. However, the appearance of low-intensity peak at *θ* = 44.5° indicates a small percentage of the (111) crystalline orientation in Ni. The XRD scan of the 2-μm-thick cathode (LSCO) film deposited on the YSZ/Ni sample by PLD first at 650°C and then at room temperature (Figure 2b) shows an LSCO (200) small broad peak at *θ* = 43°. The LSCO (100) orientation is more favorable because of its high conductivity compared to other types of crystallographic orientations [9]. The laser deposition process for the electrolyte resulted in a smooth contiguous YSZ film (Figure 3a) so as to block the hydrogen from reaching the cathode side, and for the cathode layer resulted in a cracked LSCO film as shown in Figure 3b, in order to allow the oxygen to reach the electrolyte layer during the fuel cell operation.

**Figure 4:**
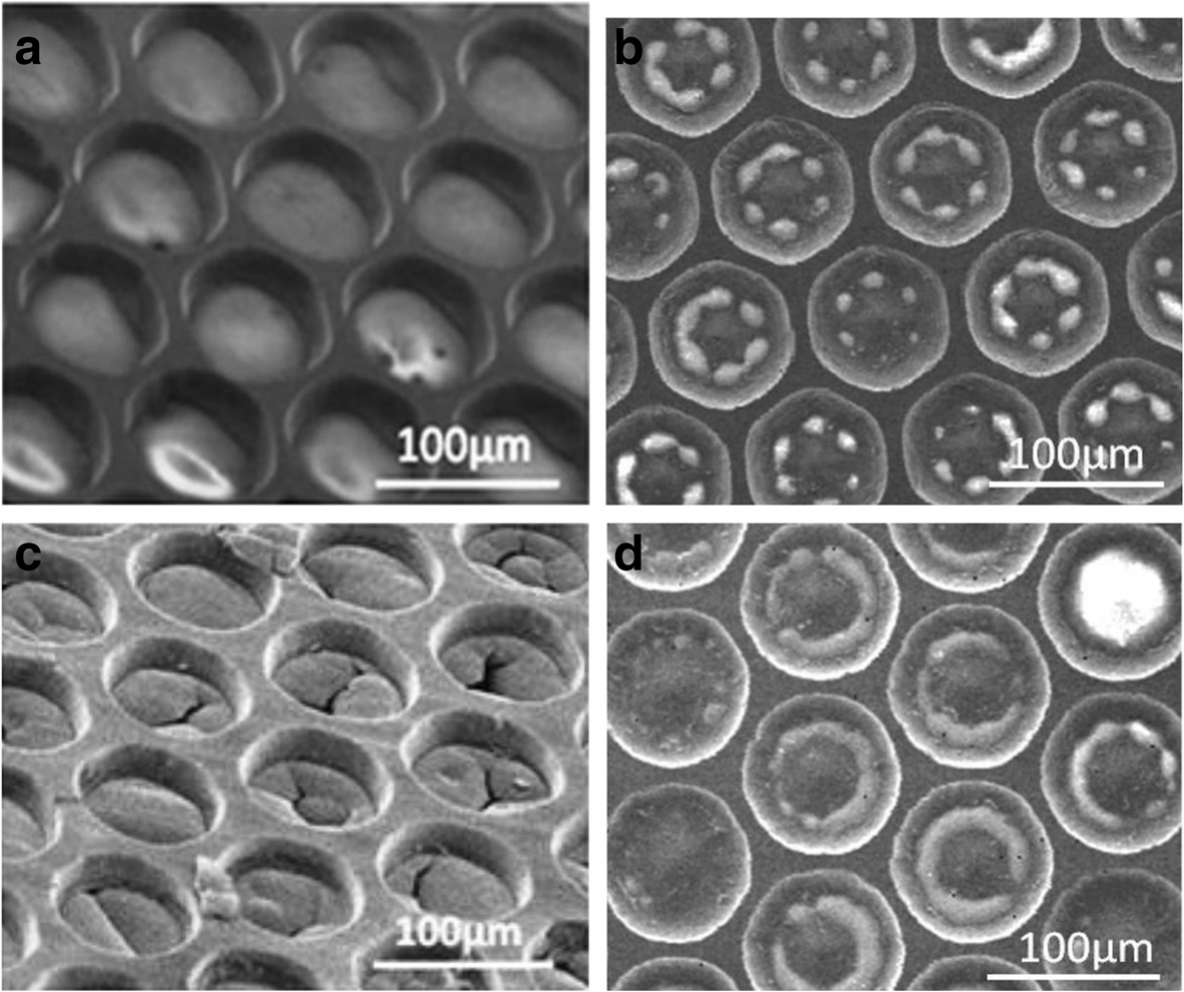
**Surface SEM micrographs from the nickel side of LSCO/YSZ/Ni cells after controlled etching on the nickel anode. (a)** Sample after wet etching, **(b)** sample after electrochemical etching, **(c)** wet-etched sample after testing at 550°C, and **(d)** electrochemically etched sample after testing at 550°C.

Since the YSZ/LSCO films were deposited on Ni foil, circular and hexagonal micropores were photolithographically patterned and etched on the nickel anodes to allow hydrogen fuel to reach the bottom electrolyte/anode interface. Both wet and electrochemical etching were tested. Wet etching was done using 0.25 M FeCl_3_ for 30 min, and electrochemical etching was done using 6 M H_2_SO_4_ for 3 min at 0.25 A and at room temperature. The SEM micrographs of these microporous openings in the nickel side of the SOFC(s) are shown in Figure 4. The sample subjected to wet etching in FeCl_3_ shows complete etching of the nickel and the pores are clean as shown in Figure 4a, and the hole size depends on the etching time. On the other hand, the sample etched electrochemically in 6 M H_2_SO_4_ exhibits incomplete etching of the nickel leaving central islands within the hexagonal frames of the pores (see Figure 4b). The islands are connected to the hexagonal frame at the middle of each side. At longer electrochemical etching time, the Ni links are lost and the middle islands always exist. In this sample, the nickel started to etch at the corners of the hexagonal frame of the photoresist. This behavior could be related to the asymmetric electric field distribution at the hexagonal corners of the photoresist frame which will be stronger in these zones because of the negative charge build up on the photoresist [10] and the etching rate of nickel due to the (SO_4_)^-2^ ions which would have higher concentrations at these zones. The islands in the hexagonal openings of the electrochemically etched pores increased the physical strength of the cell because they better support the LSCO/YSZ layers. After testing the samples for 10 h, sample with linked Ni island pores showed no cracks compared to the sample with clear pores (see Figure 4c,d). These cracks accompanied with a decrease in the cell voltage. The nickel islands also increased the surface of contact between the nickel and the YSZ, and hence, they are expected to enhance the triple-phase boundaries effect producing higher fuel cells performance.

**Figure 5:**
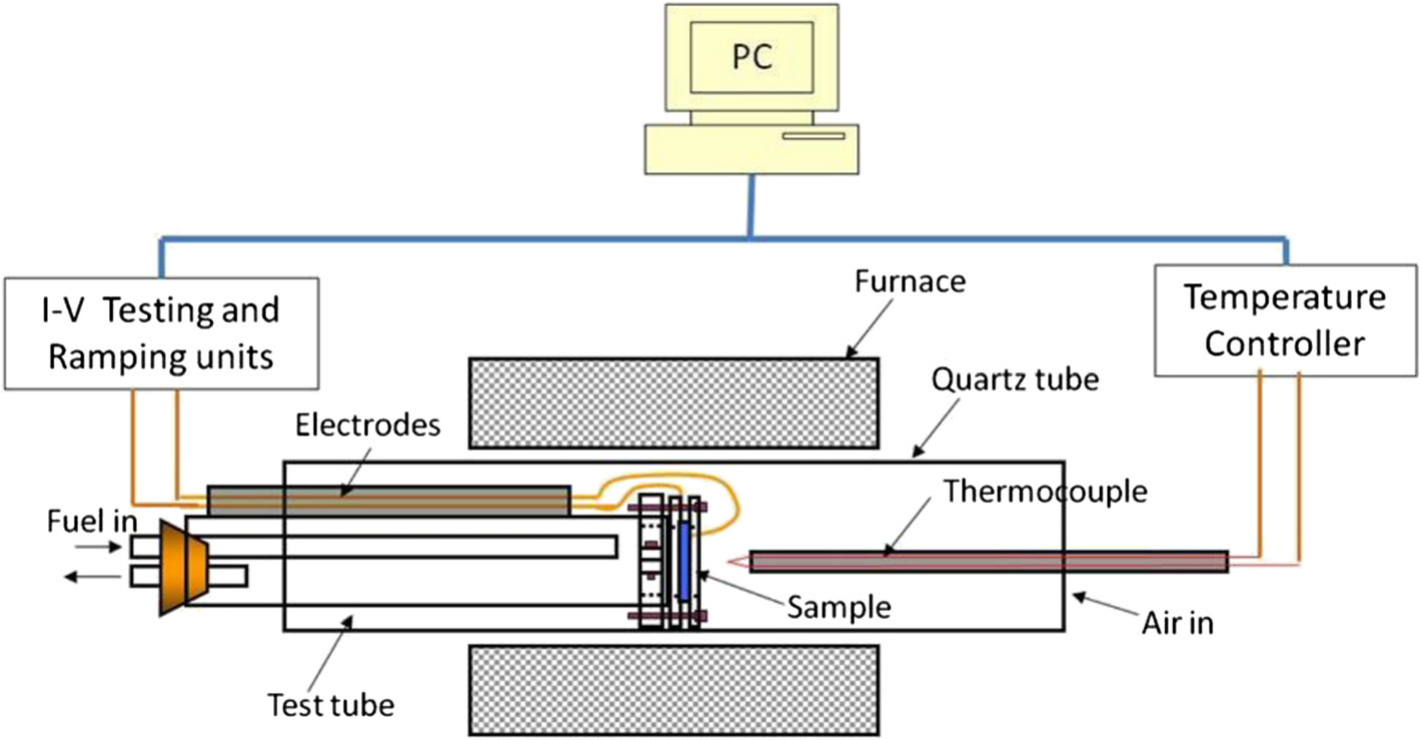
Schematic diagram for thin SOFC fuel-air test system.

**Figure 6:**
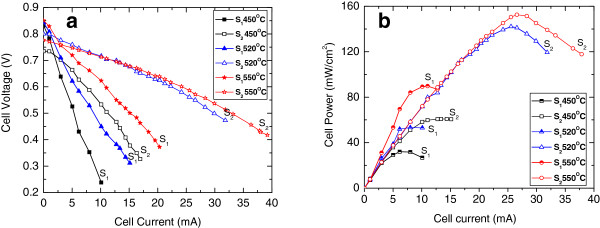
**Performance of samples etched using wet and electrochemical etching.** Performance of thin SOFC with anode clear holes (sample S_1_) and nickel islands (sample S_2_) as a function of operating temperature tested in terms of **(a)** current vs voltage and **(b)** current vs produced power.

The performance of the fabricated fuel cells was investigated using a fuel-air testing system fitted with a computer and Lab View program as shown in Figure 5. Cells were mounted in a test rig that allowed for a small feed of hydrogen to the anode side and was open to air at the cathode side. The cell was sealed into the rig by silver paste, and the test rig was heated in a programmable horizontal tubular furnace. Both *I*-*V* and electric power data have been recorded by changing the external load to the cell (0 to 2 KΩ) at fixed temperatures of 450°C, 520°C, and 550°C, at a fixed hydrogen flow. Figure 6 shows the performance of samples etched using wet and electrochemical etching. Both samples showed increases in the open circuit voltages, closed circuit current, and power density with increasing operating temperature. The sample with linked nickel islands exhibited higher closed circuit current and higher power density than the sample with clean pores. This can be related to the larger surface of contact between the Ni anode, the YSZ electrolyte, and the fuel, the triple-phase boundary which increases the oxidation process of the hydrogen at the anode and results in the release of more electrons producing higher current and thus higher power density. The areal power density of the device is lower than that of thick solid oxide fuel cells; however, due to the extreme thinness of the device, the volume power density can be much greater than thick solid oxide fuel cells, and the temperature of operation is much lower.

## Conclusions

Thin film solid oxide fuel cells were fabricated on porous nickel foils using PLD. Micropore openings were etched into the nickel foils for hydrogen fuel flow by wet and electrochemical etching so as to allow them to act as anodes. The electrochemical etching process showed incomplete etching leaving nickel islands linked to the pore frames. These islands lead to more surface area of contact between the nickel, fuel, and electrolyte - enhancement of the triple-phase boundary. The sample with the greater triple-phase boundary surface exhibits better performance and higher output power.

## Competing interests

The authors declare that they have no competing interests.

## Authors’ contributions

RE and MY carried out the sample deposition and analysis, and helped to draft the manuscript. ArI conceived of the study and participated in its design. ST and AxI conceived of the study, participated in its design and coordination, and helped to draft the manuscript. All authors read and approved the final manuscript.

## Authors’ information

Dr. RE is a senior research scientist at the Center for Advanced Materials and the Physics Department at the University of Houston. His research is focused on advanced oxide materials and also involved in materials science in the energy arena where he has contributed to work on thin film solid oxide fuel cells and to safely store the hydrogen needed for fuel cells to operate. Mr. MY is a promising research assistant at the Kazakhstan Institute for Physics and Technology and also at the Center for Advanced Materials; during his Master work, he was focusing on the development of thin film solid oxide fuel cells. Dr. ArI is the associate director of the Kazakhstan Institute for Physics and Technology and has been involved in the field of materials science for the past 10 years with focus on silicon semiconductor technology. Prof. ST is the director of the Kazakhstan Institute for Physics and Technology and is an innovator in new energy materials stemming from the application of microelectronics technologies. Besides his work in fuel cells, he also has significant efforts in novel solar cells. Prof. AxI is the director of the Center for Advanced Materials at the University of Houston where he has research programs in energy materials, computational logic materials, and materials at the physical-biological interface. He has effectively applied thin film technologies to current problems in energy including increased efficiency and reduced cost for electrochemical energy conversion.
